# Supplementary material for: Ammonia pools in zeolites for direct fabrication of catalytic centers
Source: Nat Commun. 2022 Feb 17;13:935. doi: 10.1038/s41467-022-28606-z (PMC8854602; doi:10.1038/s41467-022-28606-z)
Supplement: Supplementary file 1 — Supplementary Information [file 41467_2022_28606_MOESM1_ESM.pdf]

Supplementary Information for

Ammonia Pools in Zeolites for Direct Fabrication of Catalytic Centers

Author name:

Jie Yao[1#], Yingluo He[1#], Yan Zeng[1#], Xiaobo Feng[1,2], Jiaqi Fan[1], Shoya Komiyama[1], Xiaojing Yong[3], Wei Zhang[3], Tiejian Zhao[3], Zhongshan Guo[3], Xiaobo Peng[1,4*], Guohui Yang[1,5*], Noritatsu Tsubaki[1*]

Author affiliation:

[1] *Department of Applied Chemistry, School of Engineering, University of Toyama, Gofuku 3190, Toyama 930-8555, Japan*

[2] *Jiangsu Province Engineering Research Center of Fine Utilization of Carbon Resources, China University of Mining & Technology, Xuzhou 221116, Jiangsu, China*

[3] *National Energy Group Ningxia Coal Industry Co., Ltd., No.168 Beijing Middle Road, Yinchuan, China*

[4] *National Engineering Research Center of Chemical Fertilizer Catalyst, Fuzhou University, Fuzhou 350002, Fujian, China*

[5] *State Key Laboratory of Coal Conversion, Institute of Coal Chemistry, Chinese Academy of Sciences, Shanxi 030001, Taiyuan, China*

[*] Corresponding author, [#] These authors contributed equally to this work.

E-mail address: PENG.Xiaobo@fzu.edu.cn (Xiaobo Peng)

thomas@eng.u-toyama.ac.jp (Guohui Yang)
tsubaki@eng.u-toyama.ac.jp (Noritatsu Tsubaki)

Supplementary figures and tables

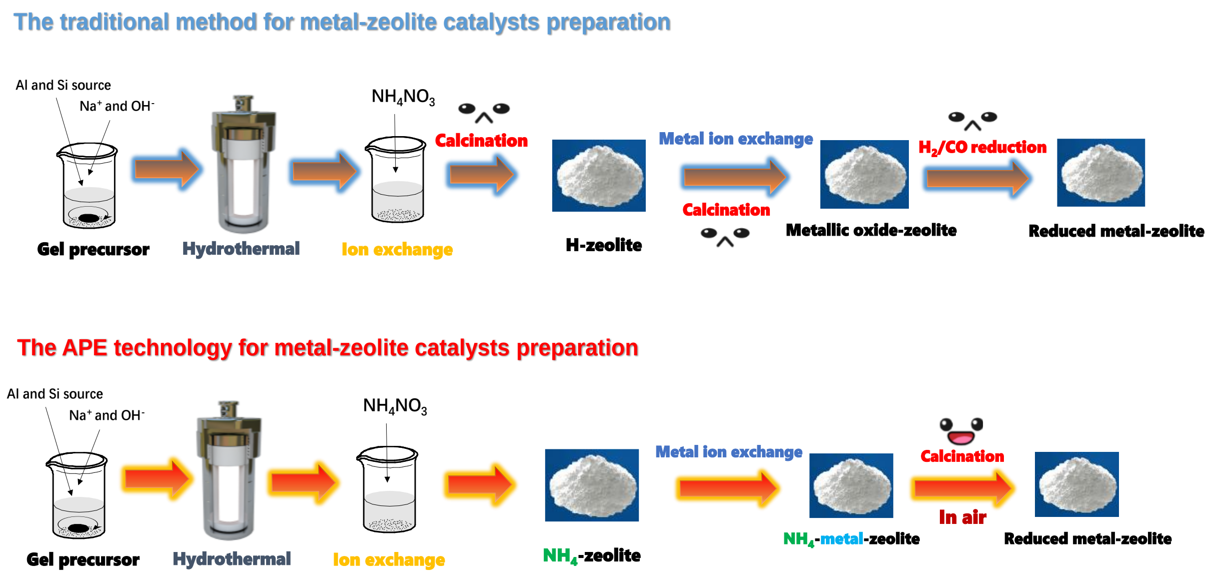

Supplementary Fig. 1 | A comparison of the processes of traditional reduction and APE technology for metal-zeolite catalysts.

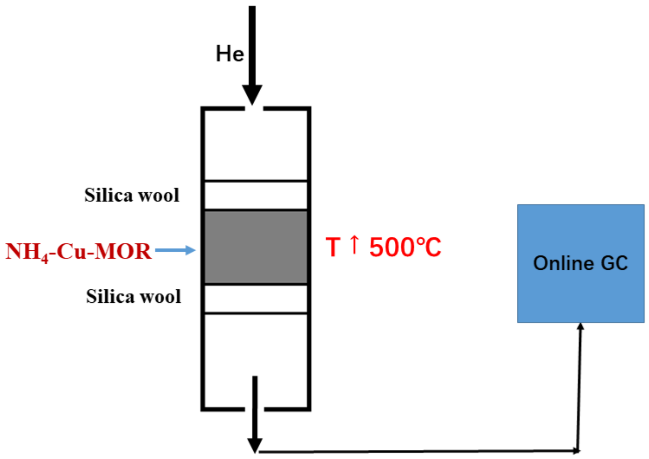

Supplementary Fig. 2 | A direct detection apparatus to analyze the effluent gas of APE reduction over the NH_4-Cu(3.41wt%)-MOR sample.

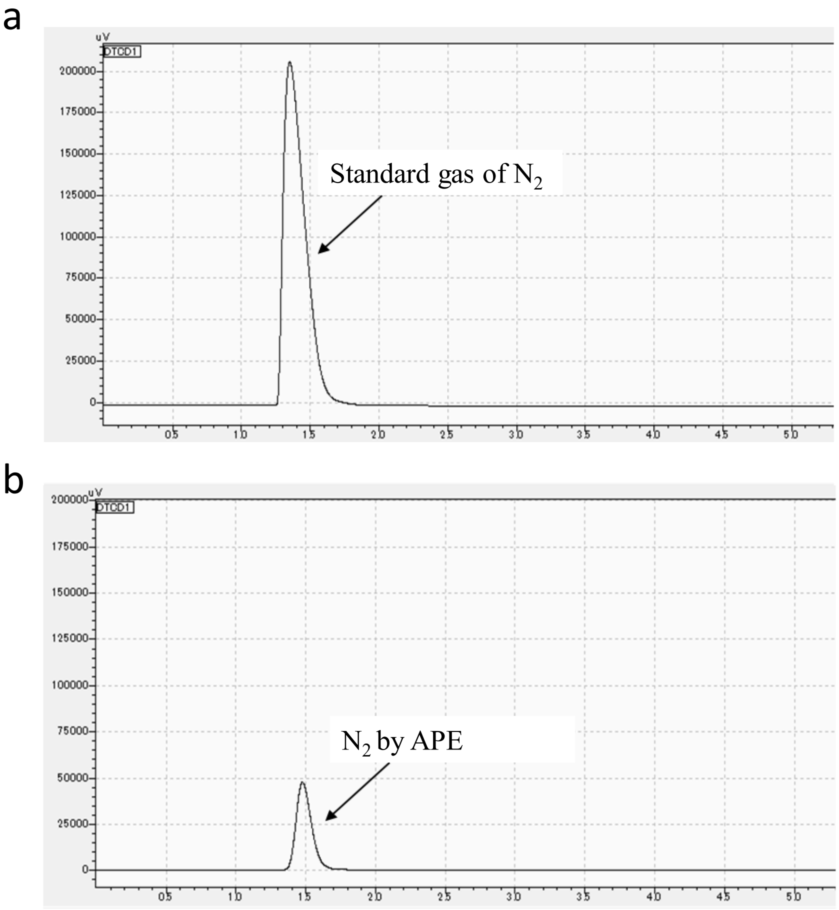

Supplementary Fig. 3 | TCD result of **a,** the N_2 peak from standard gas, and **b,** the N_2 peak from APE reduction over the NH_4-Cu(3.41wt%)-MOR sample.

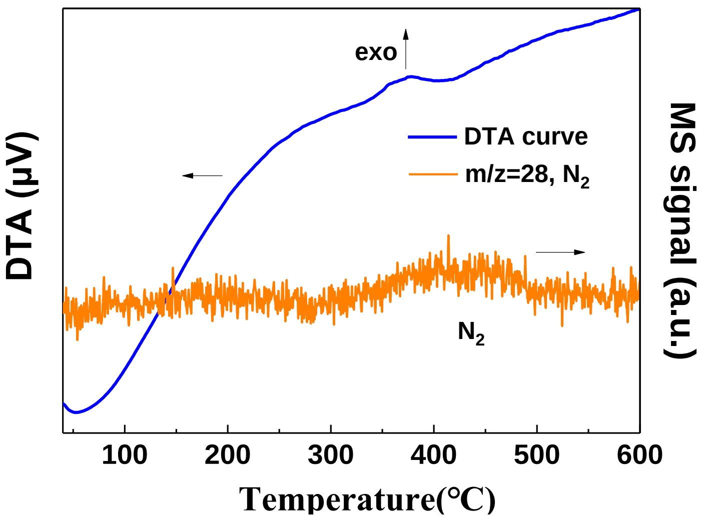

Supplementary Fig. 4 | TG-DTA-MS characterization of NH_4-Cu(3.41wt%)-MOR sample.

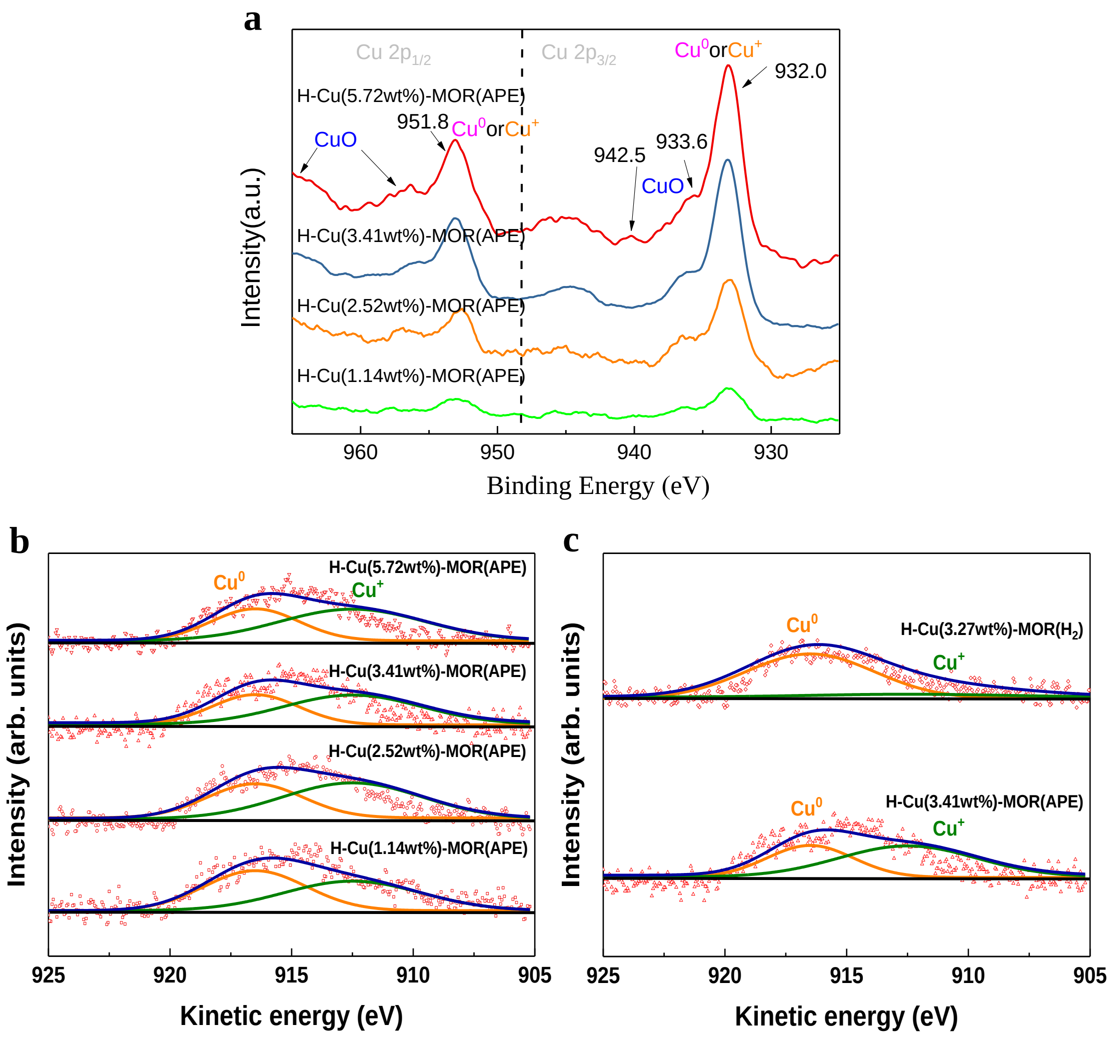

Supplementary Fig. 5 | a, The Cu *2p* XPS spectra of different H-Cu(X)-MOR(APE) samples obtained by 216 s Ar-ion etching. **b,** The Cu *LMM* Auger spectra of H-Cu(X)-MOR(APE) samples obtained at 5 nm etching depth. **c,** Comparison of the Cu *LMM* Auger spectra on H-Cu(3.41wt%)-MOR(APE) and H-Cu(3.27wt%)-MOR(H_2). The H-Cu(3.27wt%)-MOR(H_2) was prepared from Cu^{2+} ion-exchange of H-MOR zeolite, and then calcined by air and reduced via H_2. The Cu *LMM* Auger spectra disclosed that almost only Cu^0 species was formed on the H-Cu(3.27wt%)-MOR(H_2) sample, but high proportion of Cu^+ intermediate was generated on the H-Cu(X)-MOR(APE) samples.

Supplementary Table 1. The quantitative relations of different copper species in Cu-MORs samples

Catalysts	Total copper content [a] (mmol)	Ion-exchange degree [b] (%)	Percentage of different copper species [c] (%)		
			Cu^{2+}/CuO	Cu^{+}/Cu_2O	Cu^0
H-Cu(3.27wt%)-MOR(H_2)	0.026	33.6	0.0	0.0	100.0
H-Cu(3.27wt%)-MOR(Air)	0.026	33.6	100.0	0.0	0.0
H-Cu(5.72wt%)-MOR(APE)	0.045	58.4	21.2	36.1	42.7
H-Cu(3.41wt%)-MOR(APE)	0.027	35.0	13.6	40.6	45.8
H-Cu(2.52wt%)-MOR(APE)	0.020	26.0	11.1	37.2	51.7
H-Cu(1.14wt%)-MOR(APE)	0.009	11.7	9.5	35.5	55.0

[a] Determined by XRF.

[b] Calculated based on the proportion of H^+ exchanged by Cu^{2+} in total Bronsted acid sites.

[c] Determined by H_2-TPR.

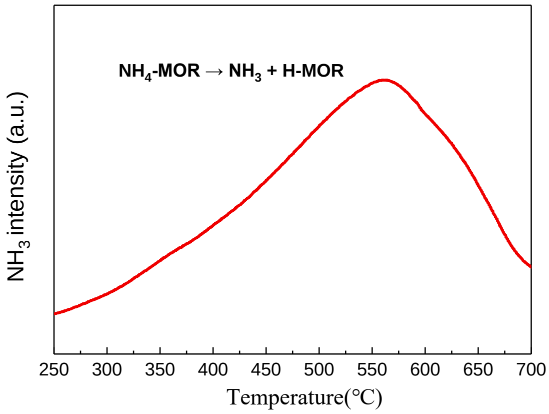

Supplementary Fig. 6 | The TPD signal of NH_4-MOR. 50 mg NH_4-MOR was loaded in the tube of TPD instrument. Pre-drying was conducted at 250 ℃ for 3h under 50 ml/min He, then the temperature programmed desorption started. The NH_3 released from the decomposition of NH_4-MOR was detected by TCD as in Supplementary Fig. 13. Through calibration titration, the total NH_3 amount from decomposition of NH_4-MOR was determined, which was equal to the total Bronsted acid content in H-MOR.

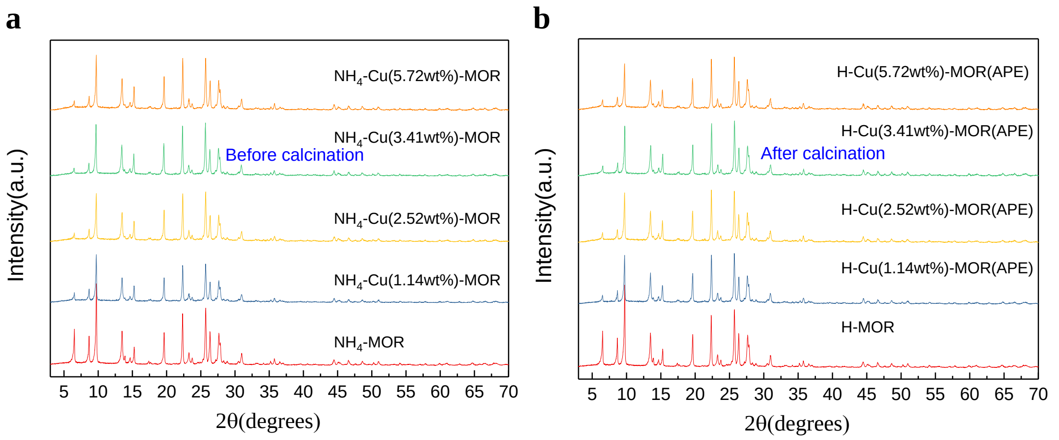

Supplementary Fig. 7 | XRD characterization of Cu-MORs samples. **a,** before APE reduction; **b,** after APE reduction.

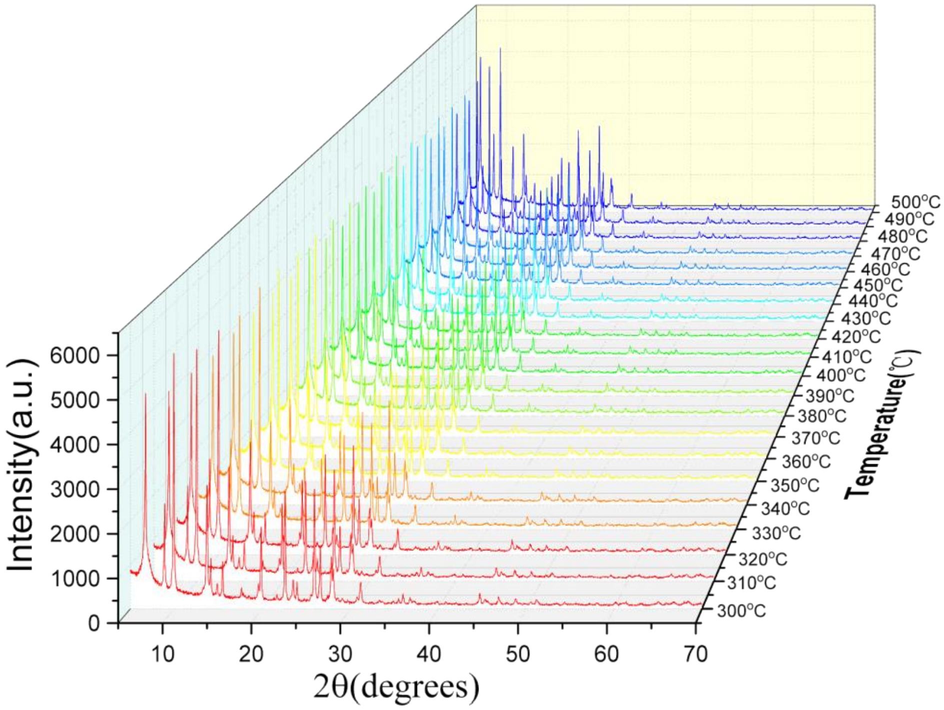

Supplementary Fig. 8 | *In-situ* XRD characterization of NH_4-Cu(3.41wt%)-MOR sample.

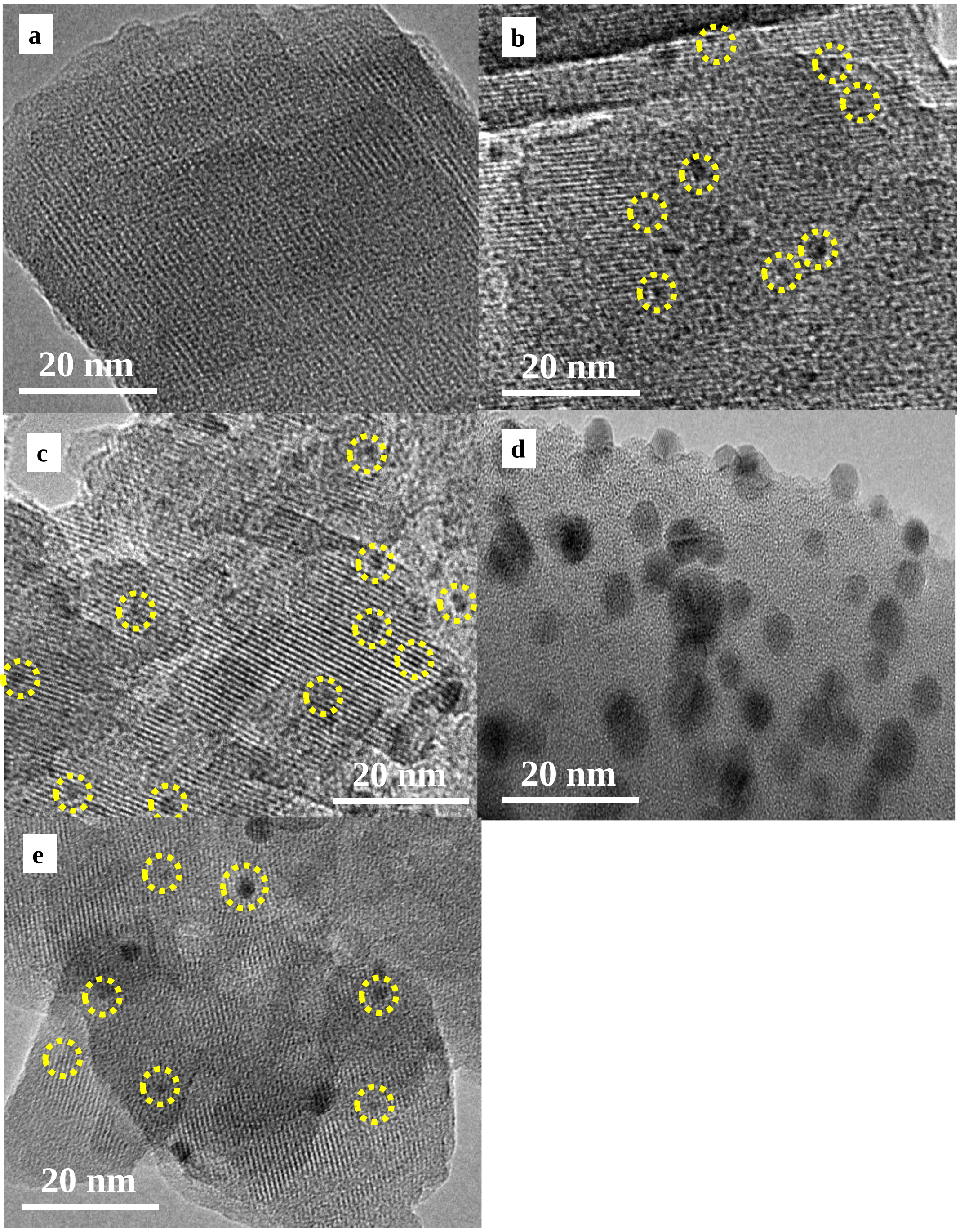

Supplementary Fig. 9 | The TEM images of **a,** Cu(1.14wt%)-MOR(APE); **b,** Cu(2.52wt%)-MOR(APE); **c,** Cu(3.41wt%)-MOR(APE); **d,** Cu(3.27wt%)-MOR(H_2); and **e,** Cu(5.72wt%)-MOR(APE).

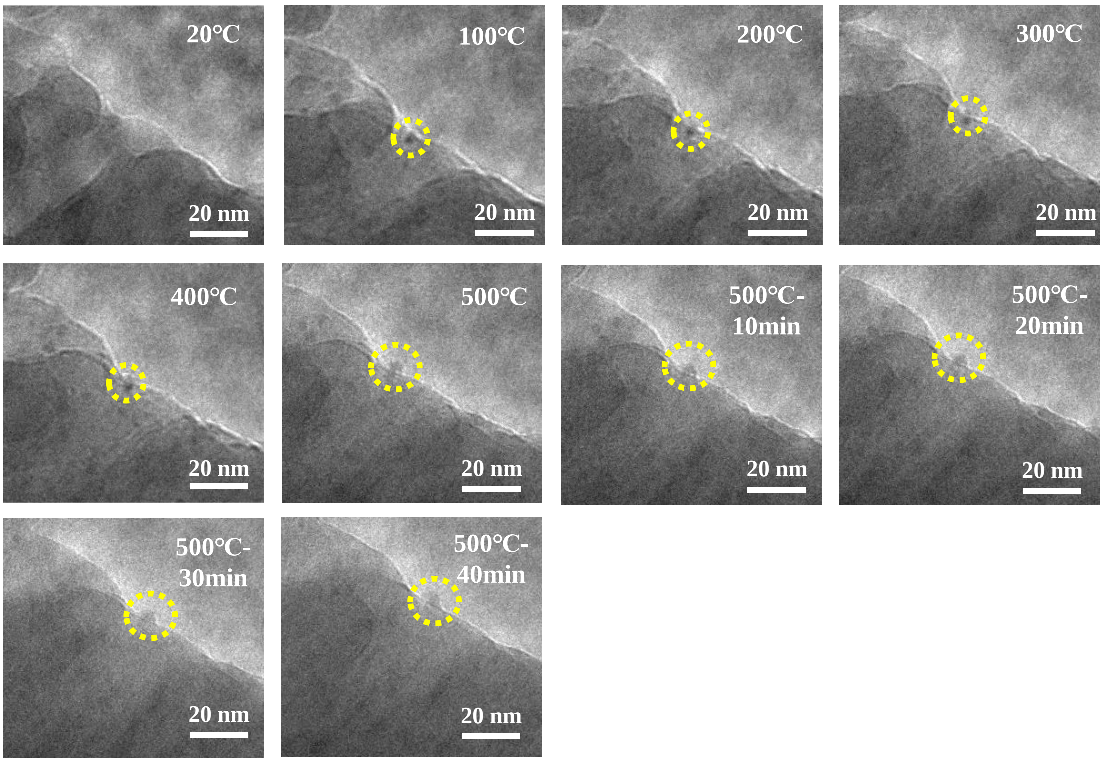

Supplementary Fig. 10 | *In-situ* TEM characterization of H-Cu(3.27wt%)-MOR(H_2) sample.

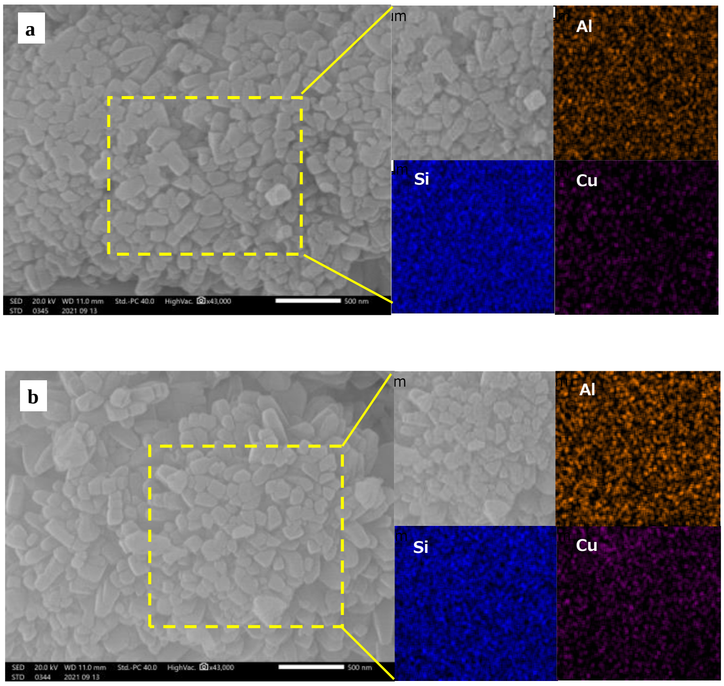

Supplementary Fig. 11 | The SEM-EDS elemental mappings of **a,** Cu(3.41wt%)-MOR(APE) and **b,** Cu(3.27wt%)-MOR(H_2).

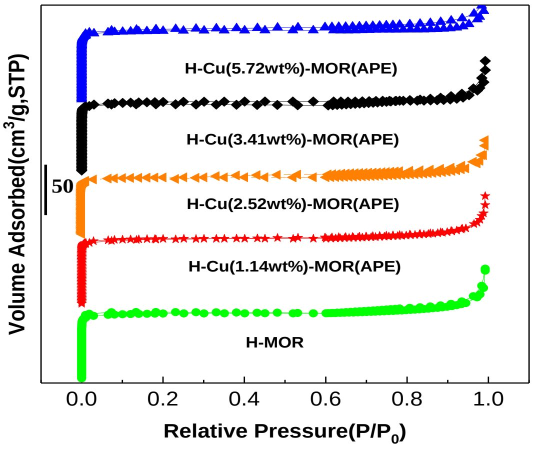

Supplementary Fig. 12 | Nitrogen adsorption and desorption isotherms of the H-MOR and H-Cu(X)-MOR(APE) samples.

Supplementary Table 2. The physicochemical properties of H-MOR and H-Cu(X)-MOR(APE) samples

Catalysts	BET surface area [a] (m^2/g)	External surface area [b] (m^2/g)	Micropore volume [c] (cm^3/g)	Mesopore volume [d] (cm^3/g)	Average pore width [e] (nm)
H-MOR	421	45	0.17	0.05	0.6
H-Cu(1.14wt%)-MOR(APE)	408	46	0.17	0.05	0.6
H-Cu(2.52wt%)-MOR(APE)	391	47	0.16	0.05	0.6
H-Cu(3.41wt%)-MOR(APE)	373	48	0.16	0.06	0.6
H-Cu(5.72wt%)-MOR(APE)	355	50	0.16	0.06	0.6

[a] Calculated at P/P_0 = 0.05-0.35

[b, c] Calculated using the t-plot method

[d] Calculated using the BJH method

[e] Evaluated by the Horvath–Kawazoe method

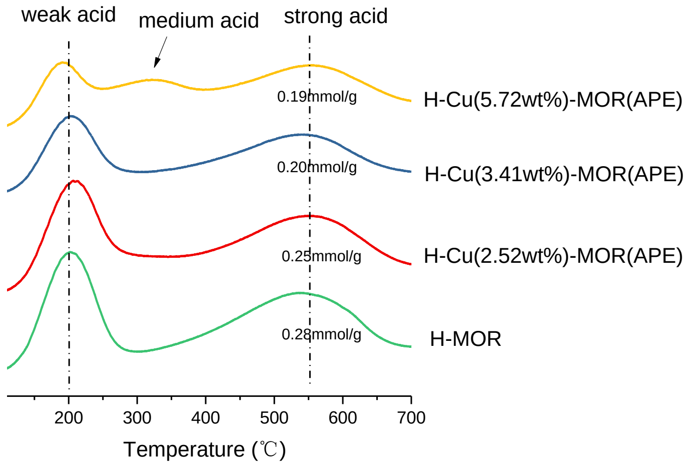

Supplementary Fig. 13 | The NH_3-TPD results of different samples.

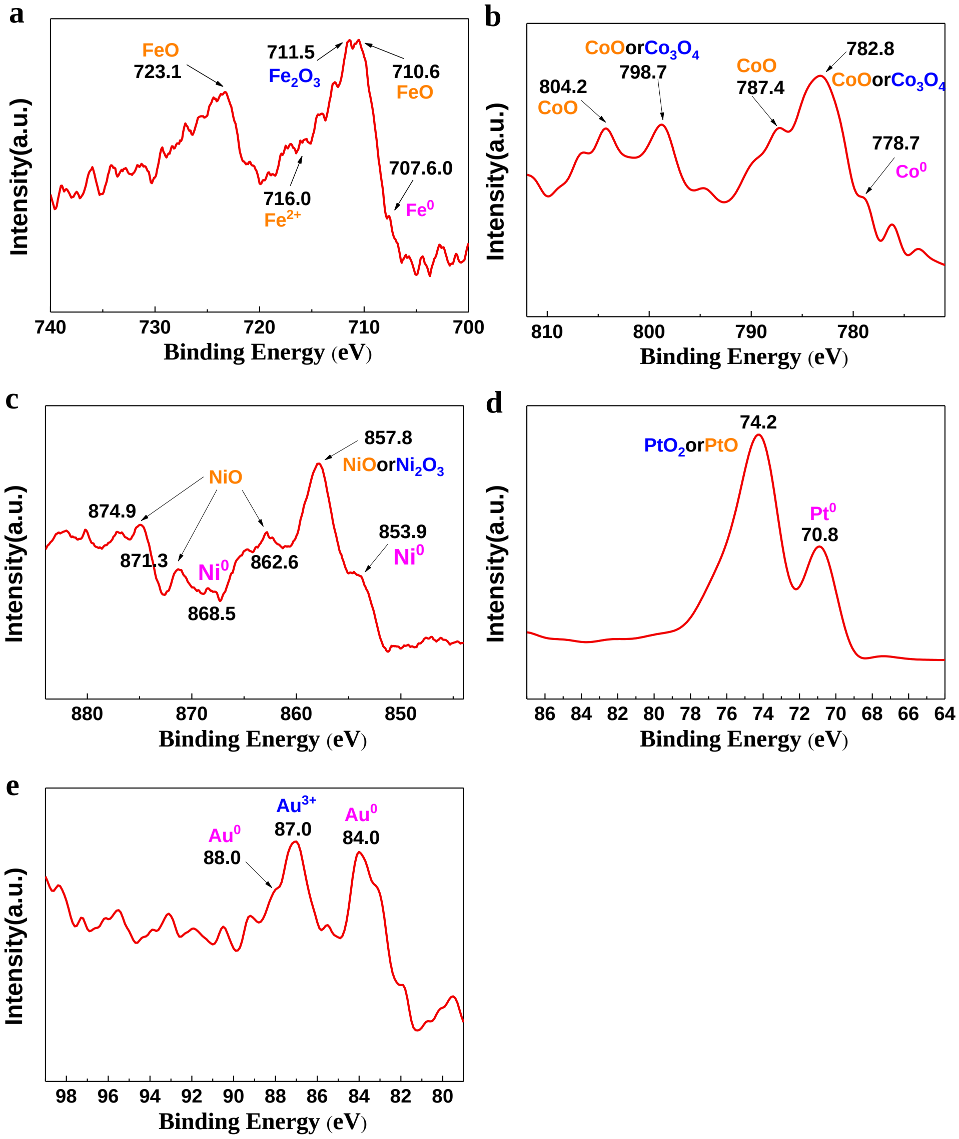

Supplementary Fig. 14 | The XPS profiles of **a,** Fe(1.8wt%)-MOR(APE); **b,** Co(0.9wt%)-MOR(APE); **c,** Ni(1.2wt%)-MOR(APE); **d,** Pt(0.9wt%)-MOR(APE); and **e,** Au(1.1wt%)-MOR(APE).

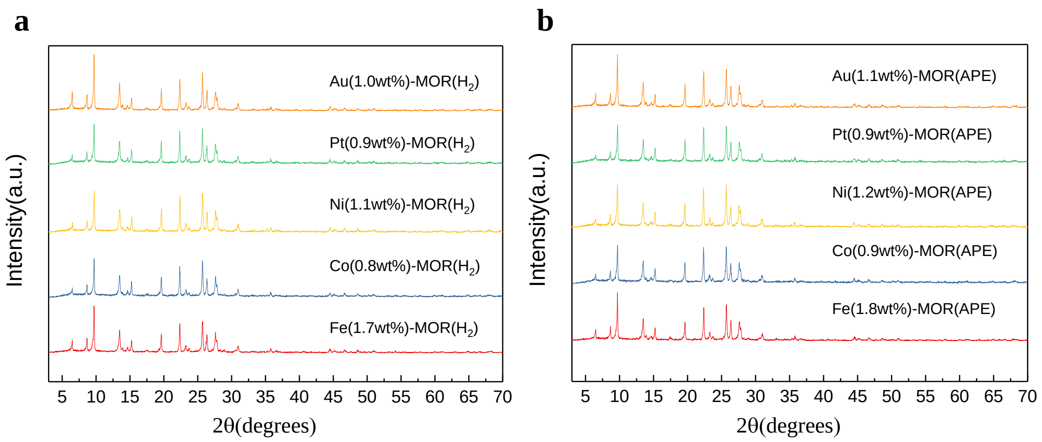

Supplementary Fig. 15 | XRD characterization of metal-MOR (metal=Fe, Co, Ni, Pt, and Au) samples fabricated by **a,** traditional H_2 reduction; and **b,** APE reduction, respectively.

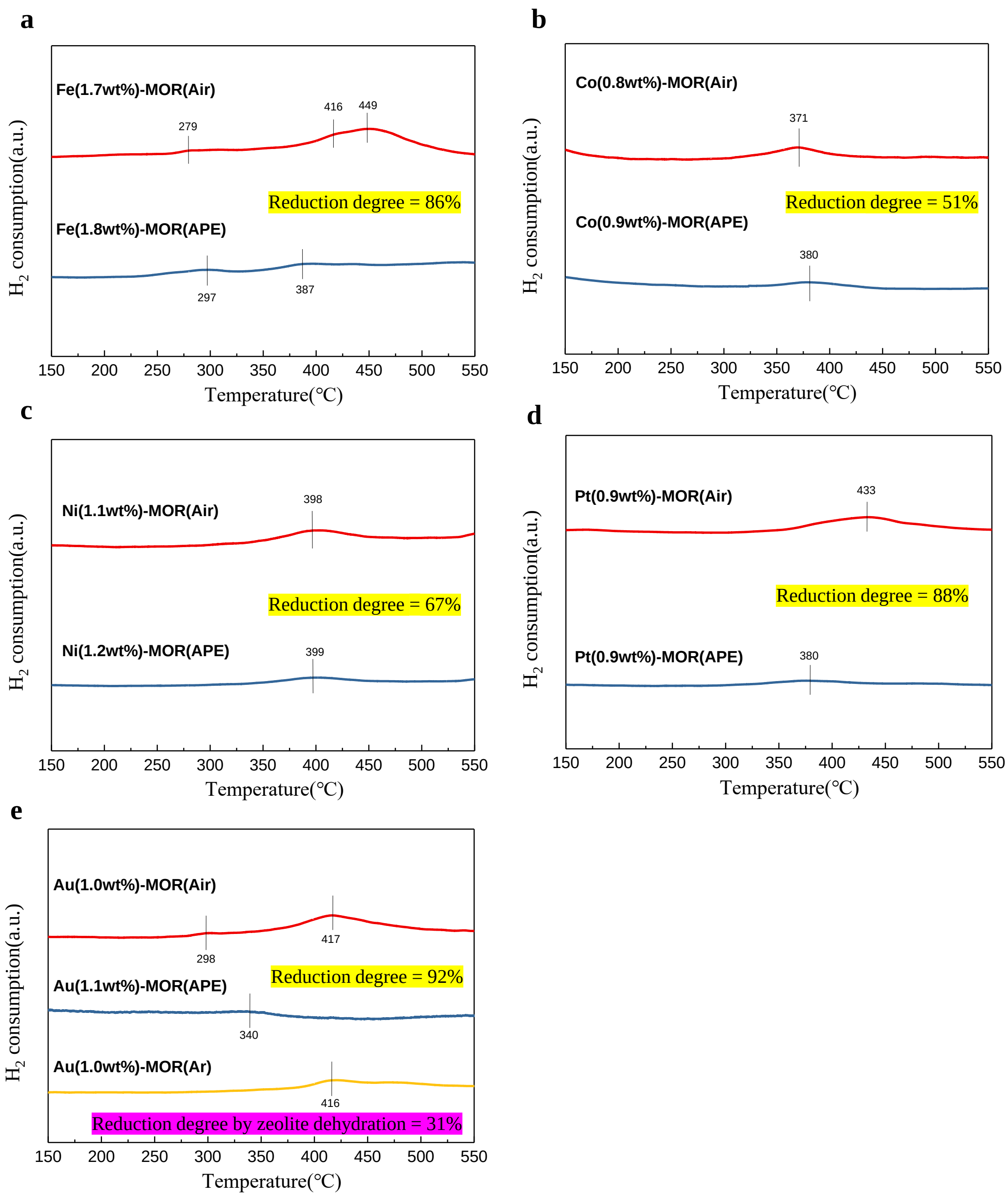

Supplementary Fig. 16 | H_2-TPR characterization of transitional metal-MOR samples and noble metal-MOR samples with or without APE reduction. **a,** Fe-MOR; **b,** Co-MOR; **c,** Ni-MOR. **d,** Pt-MOR; and **e,** Au-MOR. Note: Reduction degree = 1- (the H_2 consumption of APE sample / the H_2 consumption of the sample without reduction) (1 - (area of blue curve / area of red curve)).

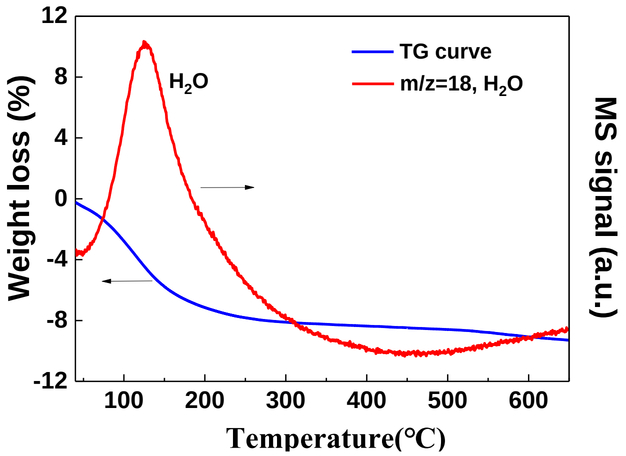

Supplementary Fig. 17 | The TG-DTA-MS result of Au(1.1wt%)-MOR sample.

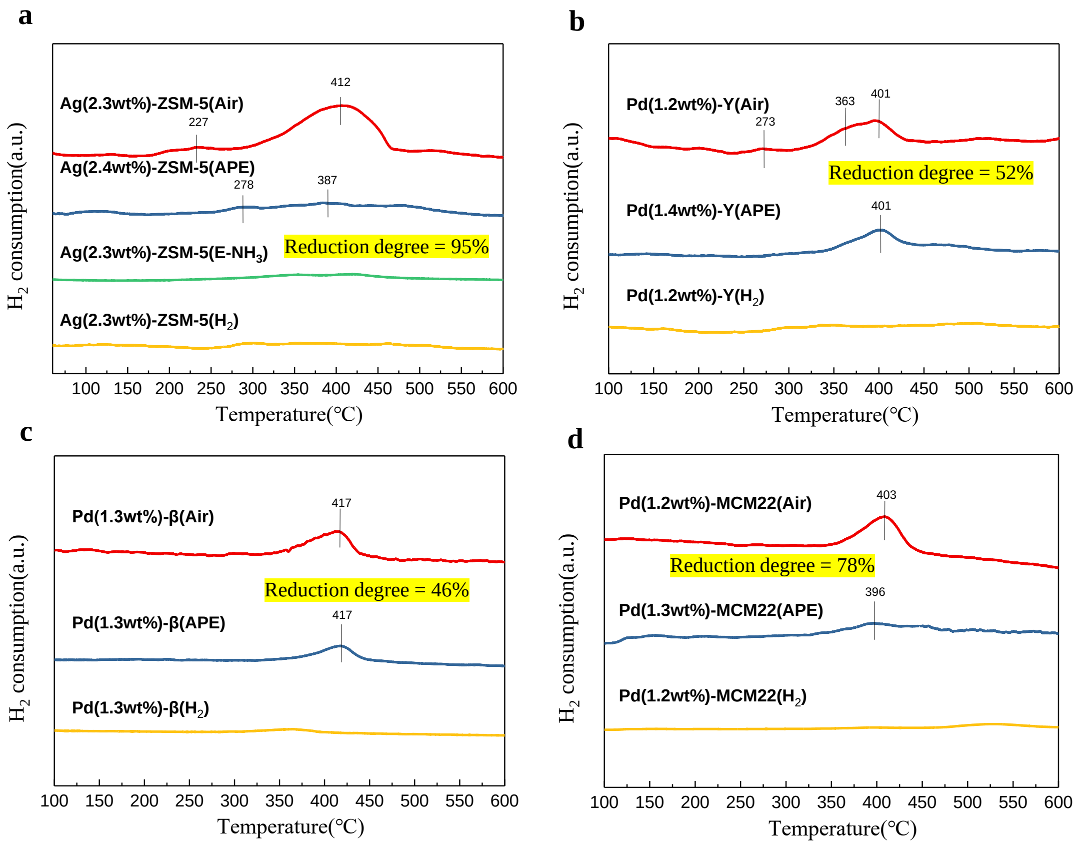

Supplementary Fig. 18 | H_2-TPR characterization of Ag-ZSM5, Pd-Y, Pd-β and Pd-MCM-22 samples with or without APE reduction. **a,** Ag-ZSM5; **b,** Pd-Y; **c,** Pd-β; and **d,** Pd-MCM-22. Note: Reduction degree = 1- (the H_2 consumption of APE sample / the H_2 consumption of the sample without reduction) (1 - (area of blue curve / area of red curve)).

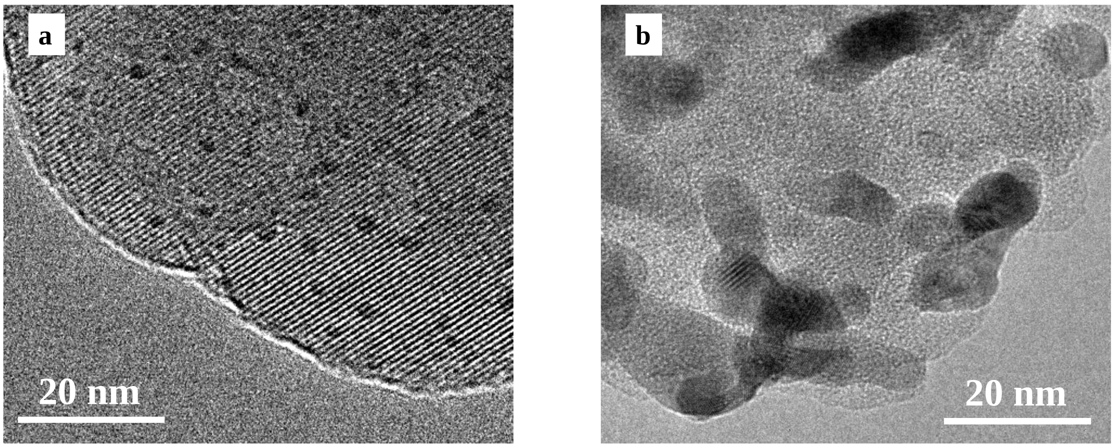

Supplementary Fig. 19 | The TEM images of **a,** Ag(2.4wt%)-ZSM5(APE) and **b,** Ag(2.3wt%)-ZSM5(H_2).

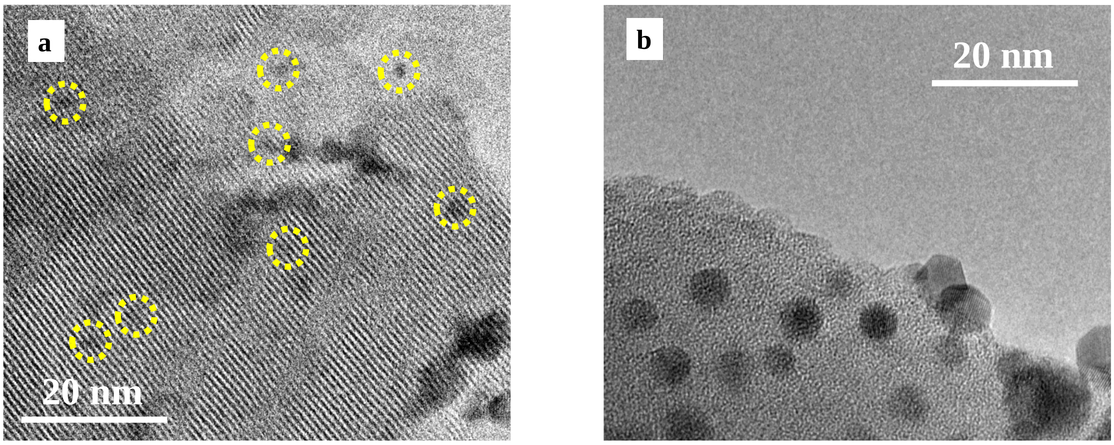

Supplementary Fig. 20 | The TEM images of **a,** Pd(1.3wt%)-β(APE) and **b,** Pd(1.3wt%)-β(H_2).

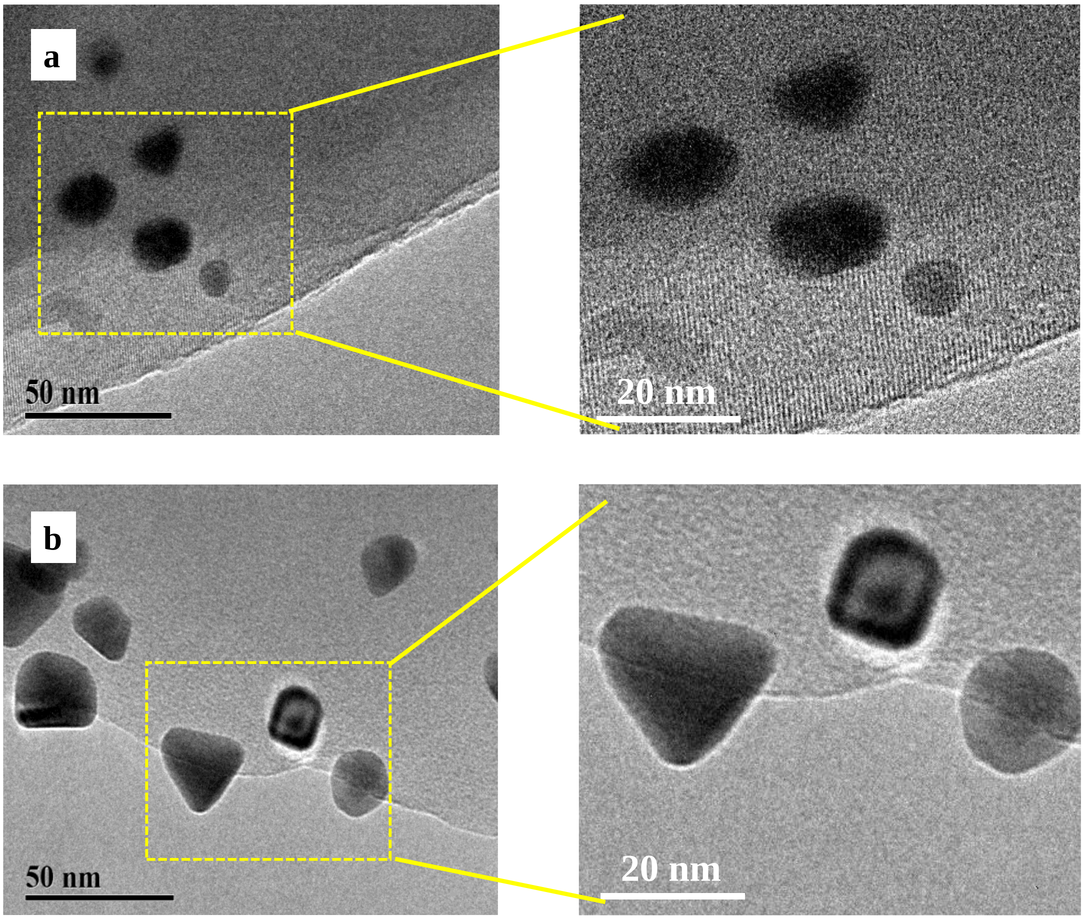

Supplementary Fig. 21 | The TEM images of **a,** Pt(0.9wt%)-MOR(APE) and **b,** Pt(0.9wt%)-MOR (H_2).

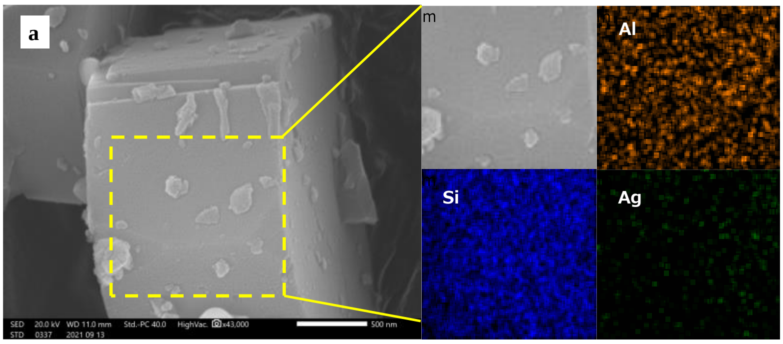

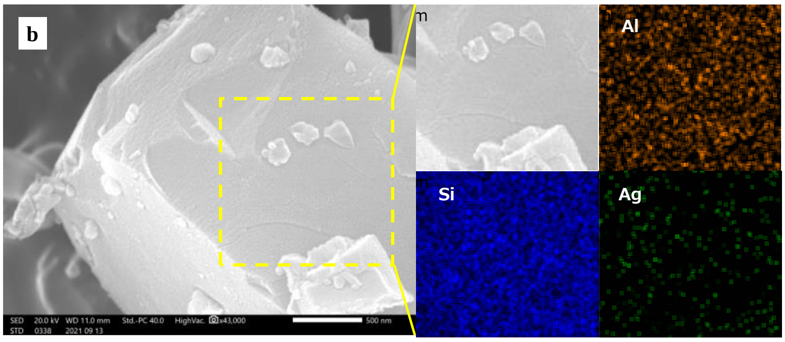

Supplementary Fig. 22 | The SEM-EDS elemental mappings of **a,** Ag(2.4wt%)-ZSM5(APE) and **b,** Ag(2.3wt%)-ZSM5(H_2).

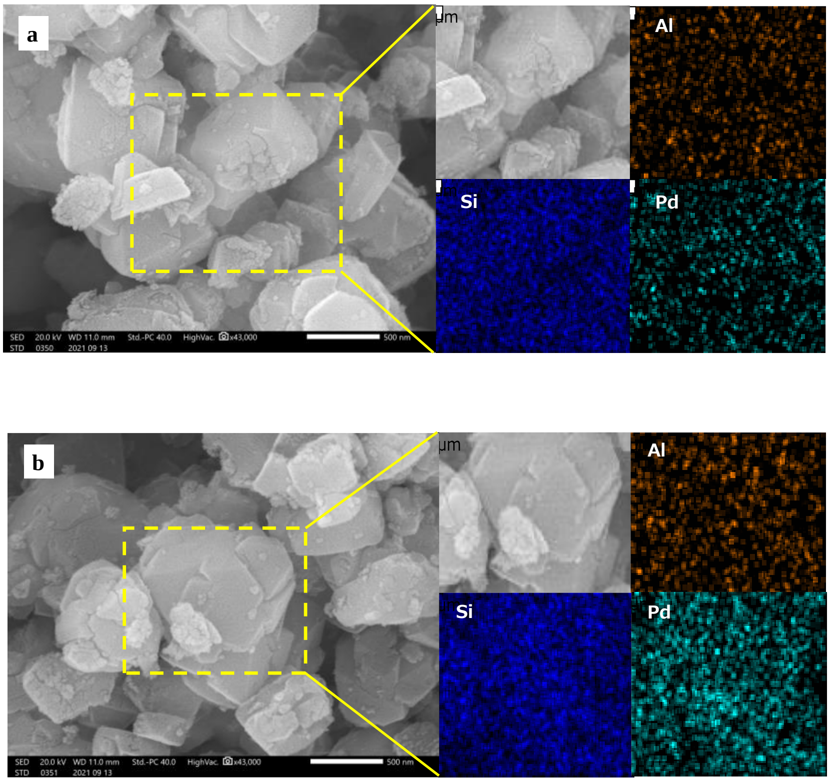

Supplementary Fig. 23 | The SEM-EDS elemental mappings of **a,** Pd(1.4wt%)-Y(APE) and **b,** Pd(1.2wt%)-Y(H_2).

Supplementary Table 3. Physical properties of parent zeolite, metal-zeolite(H_2), and metal-zeolite(APE)

Samples	Metal content [a] (wt%)	BET surface area [b] (m^2/g)
H-MOR	0	421
Cu-MOR(H_2)	2.41	401
Cu-MOR(APE)	2.52	391
H-ZSM5	0	328
Ag-ZSM5(H_2)	2.3	318
Ag-ZSM5(APE)	2.4	322
H-Y	0	586
Pd-Y(H_2)	1.2	579
Pd-Y(APE)	1.4	572

[a] Determined by XRF.

[b] Calculated at P/P_0 = 0.05-0.35.

Supplementary Table 4. The catalytic performance over H-MOR and different copper-modified MOR catalysts

Catalysts	Average conversion of DME (%)	Carbon molar selectivity (%)				
		CH_4	CO_2	MeOH	MA	Acetic acid (AA)
H-MOR	40.0	0.5	0.8	0.2	98.5	0.0
Cu^{+}(3.71wt%)-MOR	25.0	0.5	0.0	62.1	37.4	0.0
Cu^{2+}(3.35wt%)-MOR	31.7	0.7	0.0	33.6	65.7	0.0
CuO(3.21wt%)-MOR	35.0	0.1	0.0	4.9	95.0	0.0
Cu^{0}(3.27wt%)-MOR(H_2)	48.8	0.1	0.4	0.8	98.4	0.3

Reaction condition: 0.5 g catalyst, reaction temperature 220 ℃, reaction pressure 1.5 MPa, reaction gas DME/CO/Ar=4.1/92.8/3.1 (vol %), flow rate = 20 mL/min, GHSV (Gas Hour Space Velocity) = 2400 mL (g h)$^{-1}$. Note: Cu^{+}(3.71wt%)-MOR was prepared by a SSIE method with CuCl as the source of Cu^{+}; Cu^{2+}(3.35wt%)-MOR was prepared by traditional ion-exchange method then dried; CuO(3.21wt%)-MOR was prepared by traditional ion-exchange method then calcinated; and the Cu^{0}(3.27wt%)-MOR(H_2) catalyst is same with H-Cu-MOR(H_2) catalyst. The detailed production process of these catalysts is introduced in the **Methods** part.

Supplementary Table 5. The catalytic performance of different reduced Cu-MORs

Catalyst	Average conversion of DME (%)	Carbon molar selectivity (%)					STY_{MA} (mmol/(kg.h))
		CH_4	CO_2	MeOH	MA	Acetic acid (AA)	
H-Cu(1.14wt%)-MOR(APE)	51.7	0.5	0.0	0.1	99.4	0.0	2257
H-Cu(0.87wt%)-MOR(H_2)	41.5	0.3	0.0	0.1	99.6	0.0	1693
H-Cu(2.52wt%)-MOR(APE)	62.6	0.1	0.0	0.1	99.8	0.0	2744
H-Cu(2.41wt%)-MOR(H_2)	44.7	0.1	0.0	0.3	99.6	0.0	2305
H-Cu(3.41wt%)-MOR(APE)	76.7	0.7	0.0	0.5	98.5	0.3	3319
H-Cu(3.27wt%)-MOR(H_2)	48.8	0.1	0.4	0.8	98.4	0.3	2489
H-Cu(5.72wt%)-MOR(APE)	61.4	1.1	1.5	7.9	87.0	2.5	2345
H-Cu(5.45wt%)-MOR(H_2)	43.9	2.2	0.9	4.9	90.3	1.7	1853
Py-Cu(3.41wt%)-MOR(APE)[a]	50.7	0.5	0.1	1.5	94.5	3.4	2738

Reaction condition: 0.5 g catalyst, reaction temperature 220 ℃, reaction pressure 1.5 MPa, reaction time 8 h, reaction gas DME/CO/Ar=4.1/92.8/3.1 (vol %), flow rate = 20 mL/min, GHSV (Gas Hour Space Velocity) = 2400 mL $(g\ h)^{-1}$. [a] Referring to the pyridine modified catalyst. Reaction pressure 4 MPa, reaction time 100 h, reaction gas DME/CO/H_2/Ar=5.0/31.9%/60.0%/3.1 (vol %).

Supplementary Table 6. The catalytic activity of different Pd-zeolite catalysts in methane oxidation reaction

Catalysts	Product amount (μmol)				MeOH Sel. (%)	MeOH productivity (μmol/(g h))	CH_4 Conv. (‰)
	MeOH	MeOOH	HCOOH	CO/CO_2 [b]			
Pd-Y(H_2) [a]	0.12	0.00	0.00	/	100.00	8.00	0.004
Pd-Y(APE) [a]	1.12	0.00	0.00	/	100.00	74.67	0.027
Pd-MCM-22(H_2)	1.35	3.36	0.00	/	28.66	269.98	0.064
Pd-MCM-22(APE)	1.83	4.06	0.00	/	31.07	366.00	0.080
Pd-β(H_2)	0.69	1.29	0.00	/	34.85	138.01	0.027
Pd-β(APE)	0.95	1.75	0.00	/	35.19	190.03	0.037

Reaction condition: 10 mg catalyst, 0.5 M H_2O_2 in 10 mL H_2O, reaction temperature 70 ℃, reaction pressure 3.0 MPa (CH_4), reaction time 30 min.

[a] 30 mg catalyst, reaction temperature 50 ℃.

[b] No CO or CO_2 was detected in tail gas, but there may be little dissolved CO_2 in solvent.

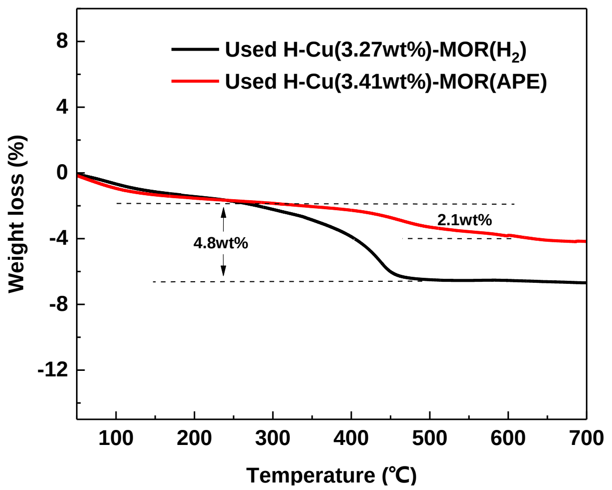

Supplementary Fig. 24 | The comparison of TG profiles of used H-Cu(3.27wt%)-MOR(H_2) and used H-Cu(3.41wt%)-MOR(APE).

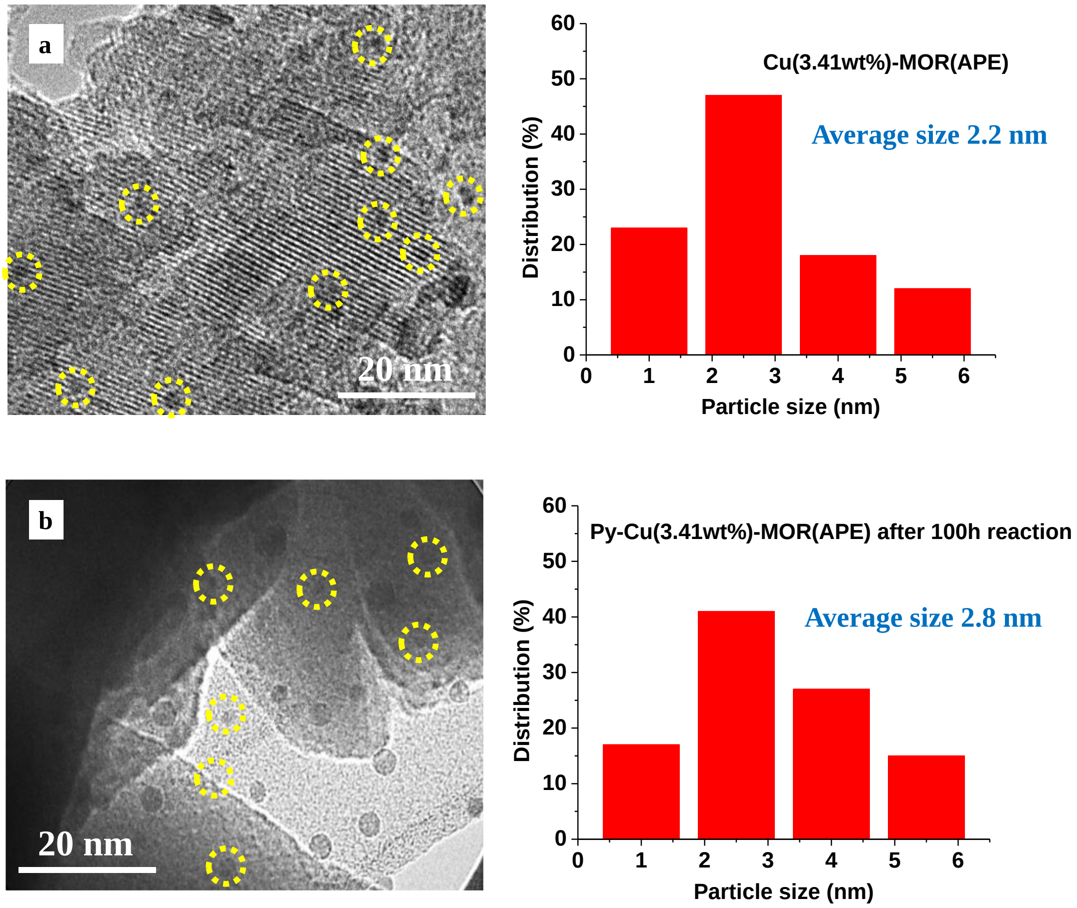

Supplementary Fig. 25 | The TEM image and copper particle size distribution of **a,** fresh Cu(3.41wt%)-MOR(APE) and **b,** collected Py-Cu(3.41wt%)-MOR(APE) after 100 h reaction.

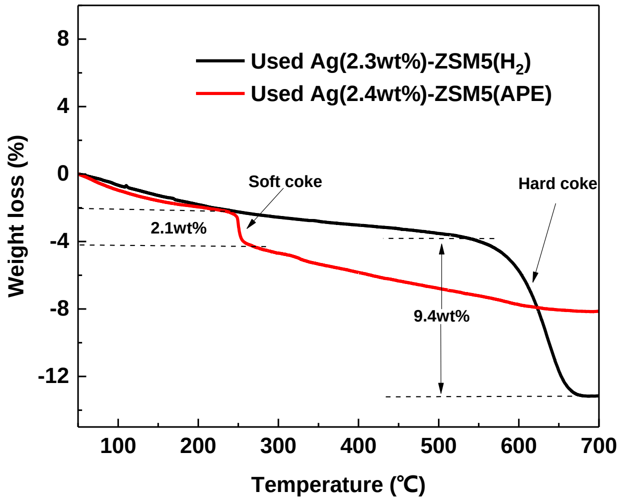

Supplementary Fig. 26 | The comparison of TG profiles of used Ag(2.3wt%)-ZSM5(H_2) and used Ag(2.4wt%)-ZSM5(APE).
